# Is a score enough? Pitfalls and solutions for AI severity scores

**DOI:** 10.1186/s41747-025-00603-z

**Published:** 2025-07-14

**Authors:** Michael H. Bernstein, Marly van Assen, Michael A. Bruno, Elizabeth A. Krupinski, Carlo De Cecco, Grayson L. Baird

**Affiliations:** 1 Department of Diagnostic Imaging, Brown Radiology Human Factors Lab, Rhode Island Hospital, Warren Alpert School of Medicine of Brown University, Providence, RI USA; 2 Department of Radiology and Imaging Sciences, Emory University, School of Medicine, Atlanta, GA USA; 3 Penn State College of Medicine, The Milton S. Hershey Medical Center, Penn State Health, Hershey, PA USA; 4 Translational Laboratory for Cardiothoracic Imaging and Artificial Intelligence, Emory University, Atlanta, GA USA

**Keywords:** Artificial intelligence, Bias, Cognition, Radiology, Reproducibility of results

## Abstract

**Abstract:**

Severity scores, which often refer to the likelihood or probability of a pathology, are commonly provided by artificial intelligence (AI) tools in radiology. However, little attention has been given to the use of these AI scores, and there is a lack of transparency into how they are generated. In this comment, we draw on key principles from psychological science and statistics to elucidate six human factors limitations of AI scores that undermine their utility: (1) variability across AI systems; (2) variability within AI systems; (3) variability between radiologists; (4) variability within radiologists; (5) unknown distribution of AI scores; and (6) perceptual challenges. We hypothesize that these limitations can be mitigated by providing the false discovery rate and false omission rate for each score as a threshold. We discuss how this hypothesis could be empirically tested.

**Key Points:**

- The radiologist-AI interaction has not been given sufficient attention.
- The utility of AI scores is limited by six key human factors limitations.
- We propose a hypothesis for how to mitigate these limitations by using false discovery rate and false omission rate.

**Graphical Abstract:**

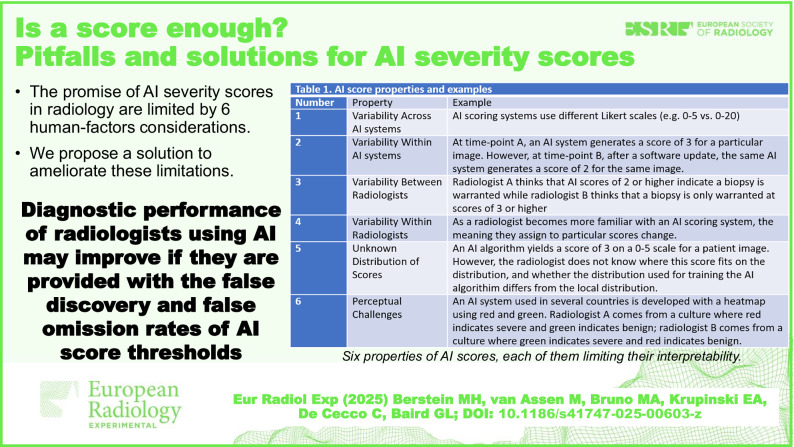

## Background

A person weighs over 80 kg. Should their primary care provider prescribe weight loss medication? A boy is 120 cm tall. Should his endocrinologist prescribe hormone treatment? Low-density lipoprotein cholesterol is above the threshold. Should the cardiologist prescribe a statin? An AI system for screening mammography assigns a severity score of 6. Should the radiologist interpreting this mammogram, and knowing the score, recommend that the patient get a diagnostic mammogram?

In these cases, more information is needed. This is second nature to us for the first two examples, but not the third. Nonetheless, when it comes to artificial intelligence (AI) scores, context remains critical. However, contextual information for severity scores (henceforth “scores”) is rarely provided. Scores refer to a quantification of the presence of a pathology. Scores have been used across a wide range of AI programs, such as those for the detection and grading of COVID-19 [1], cervical cancer [2], pulmonary nodules [3] and breast cancer [4]. How should we view a score of 2 for an AI program detecting breast cancer when scores range from 0–6? More importantly, what actions should be taken? Obviously, more information is needed.

We highlight six important human factors limitations (Table 1) inherent to the use of providing AI scores alone (*i.e*., the score in the absence of any context) that have not received attention and that undermine the intended use of AI-generated scores. We also provide a hypothesis for how these limitations can be mitigated and discuss how this hypothesis would be tested. While prior research suggests that radiologists are more accurate with the use of AI *versus* without AI [5, 6], we believe that their performance could be further enhanced by providing more context with AI scores.

**Table 1:** Artificial intelligence score properties and examples

Number	Property	Example
1	Variability across AI systems	AI scoring systems use different Likert scales (*e.g*., 0–5 *versus* 0–20).
2	Variability within AI systems	At time-point A, an AI system generates a score of 3 for a particular image. However, at time-point B, after a software update, the same AI system generates a score of 2 for the same image.
3	Variability between radiologists	Radiologist A thinks that AI scores of 2 or higher indicate a biopsy is warranted, while radiologist B thinks that a biopsy is only warranted at scores of 3 or higher.
4	Variability within radiologists	As a radiologist becomes more familiar with an AI scoring system, the meaning they assign to particular scores change.
5	Unknown distribution of scores	An AI algorithm yields a score of 3 on a 0–5 scale for a patient image. However, the radiologist does not know where this score fits on the distribution, and whether the distribution used for training the AI algorithm differs from the local distribution.
6	Perceptual challenges	An AI system used in several countries is developed with a heatmap using red and green. Radiologist A comes from a culture where red indicates severe and green indicates benign; radiologist B comes from a culture where green indicates severe and red indicates benign.

## Limitations of AI scores

### Limitation 1. Variability across AI systems

The method used for generating scores in particular AI algorithms is often proprietary. Further, each company has developed its own scoring system from its own training data, and, therefore, the meaning of scores and scoring systems is not universal. For instance, one AI program (Mammoscreen) for screening mammography has scores that range from 1 (lowest suspicion) to 10 (highest suspicion) [7] while another (iCAD) provides scores based on percents that presumably range from 0 to 100 [8]. Thus, scoring methods vary even among algorithms targeted to the same pathology. This issue becomes even more acute when comparing scores generated by algorithms working across different pathologies and data sources (*e.g*., acquisition devices). This lack of consistency will be cognitively demanding for radiologists; they will be barraged with multiple different scoring systems in the course of a single day. They may even be presented with multiple different scoring systems for an individual patient if there are several AI algorithms used, each for the detection of a different pathology. The lack of consistency between systems is likely to exacerbate confusion and increase the risk of poor decision-making and may engender alarm fatigue [9] and contribute to a higher cognitive load [10]; multiple alarms will be sounding, but the alarms will not even have the same meaning in terms of severity or urgency.

### Limitation 2. Variability within AI systems

Scoring systems are not only inconsistent from one AI system to another but are also inconsistent for the same AI system over time. Imagine an AI system for breast cancer detection. One year later, the system is updated based on analysis of prior results, changing the meaning of AI-generated scores. The scoring system before the update and the scoring system after the update will differ. We can expect this to be particularly likely in European Union (EU) countries, because the EU AI Act mandates postmarket monitoring for high-risk AI systems [11].

Consider the following hypothetical: an AI system is released with scores ranging from 0 to 5. When the system is updated 1 year later, it becomes less conservative due to a high proportion of false positives. Thus, does a score of 3 before the update mean the same as a score of 3 after the update? How should a radiologist react to images that formerly had a score of 3 but now have a score of 2? Will radiologists even be aware of an update occurring, and how the update has changed the scores? This also invites the question of whether patients and radiologists should have access to both scores.

### Limitation 3. Variability between radiologists

Radiologists will likely interpret the same AI score for the same case differently, just as the interpretation of images without AI also varies [12, 13]. For example, some may think that a biopsy is typically warranted any time the AI score is 2 or 3 or higher, while others may think the same score does not usually warrant a biopsy. Clinical decision-making can vary between two family medicine physicians interpreting the same lab value for the same patient; likewise, two radiologists can differ in their interpretation of the same AI score for the same case. Any score, perhaps excluding the lowest and the highest ones, has no inherent interpretation, even though radiologists will implicitly assign meaning to it, and likely in inconsistent ways. Without clear guidance regarding what a score means, radiologists will be especially vulnerable to heuristics and cognitive biases, such as anchoring bias [14], recency and primacy effects [15] and automation bias [16].

### Limitation 4. Variability within radiologists

Radiologists’ perception of AI scores will likely be changing within themselves over time. We should anticipate a learning effect such that the benefit of using a scoring system might be weakest for a radiologist when they first begin using that system, or *vice versa*. The extent to which a radiologist will rely on AI output is also likely fluid, as increased experience could enhance or diminish automation bias, which prior work has shown radiologists are vulnerable to [16]. In addition, there may also be a general experience effect where radiologists’ perception of scores changes over time with increased exposure and feedback, especially for residents or junior radiologists. Furthermore, as radiologists’ perception of AI scores changes over time, the AI scores themselves will also be changing over time (see limitation 2). Thus, radiologists’ perceptions of AI scores will lag behind an ever-changing AI system.

### Limitation 5. Unknown distribution of AI scores

AI scores exist on a distribution relating to pathology. However, without explicitly knowing what that distribution is, and where a patient’s observation fits on that distribution, radiologists’ implicit perceptions of the distribution will be used instead. What is more, the distribution used in training and even validation does not necessarily generalize to the distribution for a particular clinical setting. This is because the training distribution and the local distribution may reflect different populations, including a different base rate of pathology. The point here is that the interpretation of a score alone is very unclear without knowing where that score lies on the distribution—that is, the score alone is not enough information.

### Limitation 6. Perceptual challenges

Some scoring systems rank severity visually, by display color. For example, red is “severe,” green is “not severe,” and yellow is in the middle. However, color is not necessarily universal in meaning, and a color scheme created in one culture may not translate to another [17]. Also red and green are often used despite the fact 8% of men and 0.4% of women [18] who are European Caucasians have red-green colorblindness. Heatmaps can be an excellent representation of the distribution behind a given AI score for a given patient, but they should be adjusted using different colors of the radiologist’s choosing, or at minimum, a color legend should always be provided. Perceptual variability could be increased by the fact that different display monitors can be calibrated to different color scales and that, as these monitors degrade over time, the colors can change.

## Hypothesized mitigation

Fundamentally, the limitations we described above rest on the issue that there is no shared meaning across (and within) radiologists and AI algorithms regarding how AI scores should be interpreted. There are solutions available to help address these issues.

As we have discussed elsewhere [19], when an AI score is presented to a radiologist, the false discovery rate (FDR) (1 minus positive predictive value) and false omission rate (FOR) (1 minus negative predictive value) corresponding with that score as a threshold should also be provided. The FDR refers to the rate at which a given AI score or higher is applied to a normal examination (a false positive), and the FOR refers to the rate at which a value lower than the AI score under consideration is applied to an examination harboring the searched pathology (a false negative). These rates can be estimated by first running an AI algorithm on local historical data, then evaluating the relationship between known pathology and the AI scores by calculating both FDR and FOR for each score as a threshold. We recommend using local historical data (whenever possible), rather than estimates provided by the AI company, because the populations may differ. As such, calculating the FDR and FOR from the sensitivity and specificity provided by the AI company may not be generalizable to a local population, even after adjusting for the local prevalence rate. These FDR and FOR values can then be used as a reference for local radiologists’ interpretation of AI scores of future cases. Importantly, reporting FDR and FOR values for each threshold of a score, as we propose, is different from reporting the overall FDR and FOR for the AI system.

For example, if an AI algorithm for breast cancer ranges from 0 to 1 and a radiologist knows that an AI score of 0.2 or higher translates to an FDR of 98% and an AI score below 0.2 translates to an FOR of 0.24% when applied to a sufficiently large local case series, then a radiologist is equipped to better evaluate the risk of breast cancer for a mammogram with that score. Conversely, providing the score with no such contextual information for the local population makes it uninterpretable concerning cancer risk, other than the obvious of higher scores having greater risk. Such reporting would be consistent with the manner in which the EU AI Act focuses on increased transparency for AI systems [11].

Therefore, we hypothesize that providing radiologists with the corresponding FDR and FOR for AI scores, the radiologist inter- and inter-rater reliability will increase relative to providing then with AI scores alone. In addition, we hypothesize that by providing (*versus* not providing) radiologists with FDR and FOR alongside AI scores, diagnostic performance will be improved (especially in reducing false positives). These hypotheses can be tested using a fully-crossed multicase multireader design where radiologists interpret a series of identical images under two different conditions with a washout period (*e.g*., 30 days) to control for a carryover effect, counterbalanced by order. In one condition, radiologists would evaluate cases with an AI score but with no FDR and FOR information. Conversely, for the other condition, radiologists would evaluate the same cases with the same AI scores but with the corresponding FDR and FOR.

Radiologists should be selected randomly as a sample (representative of the population of radiologists). Fleiss κ would be used to quantify inter- and intra-rater reliability, while the true positive, true negative, false positive, and false negative rates would be evaluated with a generalized linear mixed model assuming a binary distribution.

## Conclusions

AI severity scores represent an important attempt to provide radiologists and other physicians with a clear, quantitative indication of the likelihood or probability of pathology. However, there are several limitations with their use as currently used. We hypothesize that these limitations can be mitigated by providing FDR and FOR values for the AI scores, as a threshold.

## Abbreviations

AI: Artificial intelligence
FDR: False discovery rate
FOR: False omission rate

## References

[1] Lessmann N, Sánchez CI, Beenen L et al (2021) Automated assessment of COVID-19 reporting and data system and chest CT severity scores in patients suspected of having COVID-19 using artificial intelligence. Radiology 298:E18–E28. 10.1148/radiol.2020202439 32729810 10.1148/radiol.2020202439 PMC7393955

[2] Bao H, Sun X, Zhang Y et al (2020) The artificial intelligence‐assisted cytology diagnostic system in large‐scale cervical cancer screening: a population‐based cohort study of 0.7 million women. Cancer Med 9:6896–6906. 10.1002/cam4.3296 32697872 10.1002/cam4.3296 PMC7520355

[3] Baldwin DR, Gustafson J, Pickup L et al (2020) External validation of a convolutional neural network artificial intelligence tool to predict malignancy in pulmonary nodules. Thorax 75:306–312. 10.1136/thoraxjnl-2019-214104 32139611 10.1136/thoraxjnl-2019-214104 PMC7231457

[4] Dembrower K, Wåhlin E, Liu Y et al (2020) Effect of artificial intelligence-based triaging of breast cancer screening mammograms on cancer detection and radiologist workload: a retrospective simulation study. Lancet Digit Health 2:E468–E474. 10.1016/S2589-7500(20)30185-0 33328114 10.1016/S2589-7500(20)30185-0

[5] Lee JH, Kim KH, Lee EH et al (2022) Improving the performance of radiologists using artificial intelligence-based detection support software for mammography: a multi-reader study. Korean J Radiol 23:505–516. 10.3348/kjr.2021.0476 35434976 10.3348/kjr.2021.0476 PMC9081685

[6] Ahn JS, Ebrahimian S, McDermott S et al (2022) Association of artificial intelligence-aided chest radiograph interpretation with reader performance and efficiency. JAMA Netw Open 5:e2229289. 10.1001/jamanetworkopen.2022.29289 36044215 10.1001/jamanetworkopen.2022.29289 PMC9434361

[7] MammoScreen. Available via https://www.mammoscreen.com/the-score. Accessed 9 Jan 2025

[8] Letter H, Peratikos M, Toledano A et al (2023) Use of artificial intelligence for digital breast tomosynthesis screening: a preliminary real-world experience. J Breast Imaging 5:258–266. 10.1093/jbi/wbad015 38416890 10.1093/jbi/wbad015

[9] Ash JS, Sittig DF, Campbell EM, Guappone KP, Dykstra RH (2007) Some unintended consequences of clinical decision support systems. AMIA Annu Symp Proc 2007:26–30 PMC2813668 18693791

[10] Sweller J (2011) Cognitive load theory. Psychol Learn Motiv 55:37–76. 10.1016/B978-0-12-387691-1.00002-8

[11] European Parliament and the Council of the European Union (2024) Regulation (EU) 2024/1689 of 13 June 2024 laying down harmonised rules on artificial intelligence and amending regulations (EC) no 300/2008, (EU) no 167/2013, (EU) no 168/2013, (EU) 2018/858, (EU) 2018/1139 and (EU) 2019/2144 and directives 2014/90/EU, (EU) 2016/797 and (EU) 2020/1828. OJ 1–144. accessed May 1 2025 Available via https://eur-lex.europa.eu/legal-content/EN/TXT/PDF/?uri=OJ:L_202401689

[12] van Assen M, Zandehshahvar M, Maleki H et al (2022) COVID-19 pneumonia chest radiographic severity score: variability assessment among experienced and in-training radiologists and creation of a multireader composite score database for artificial intelligence algorithm development. Br J Radiol 95:20211028. 10.1259/bjr.20211028 35451863 10.1259/bjr.20211028 PMC10996404

[13] Lee AY, Wisner DJ, Aminololama-Shakeri S et al (2017) Inter-reader variability in the use of BI-RADS descriptors for suspicious findings on diagnostic mammography: a multi-institution study of 10 academic radiologists. Acad Radiol 24:60–66. 10.1016/j.acra.2016.09.010 27793579 10.1016/j.acra.2016.09.010

[14] Furnham A, Boo HC (2011) A literature review of the anchoring effect. J Socio Econ 40:35–42. 10.1016/j.socec.2010.10.008

[15] Murre JM, Dros J (2015) Replication and analysis of Ebbinghaus’ forgetting curve. PLoS One 10:e0120644. 10.1371/journal.pone.0120644 26148023 10.1371/journal.pone.0120644 PMC4492928

[16] Bernstein MH, Atalay MK, Dibble EH et al (2023) Can incorrect artificial intelligence (AI) results impact radiologists, and if so, what can we do about it? A multi-reader pilot study of lung cancer detection with chest radiography. Eur Radiol 33:8263–8269. 10.1007/s00330-023-09747-1 37266657 10.1007/s00330-023-09747-1 PMC10235827

[17] Aslam MM (2006) Are you selling the right colour? A cross‐cultural review of colour as a marketing cue. J Mark Commun 12:15–30. 10.1080/13527260500247827

[18] Birch J (2012) Worldwide prevalence of red-green color deficiency. J Opt Soc Am A Image Sci Vis 29:313–320. 10.1364/JOSAA.29.000313 10.1364/JOSAA.29.000313 22472762

[19] Chung M, Bernstein MH, Yala A, Baird GL (2025) A universal translator for AI scores: providing context using error. Preprint at 10.1101/2025.02.28.25323066

